# Practical guidelines for developing digital health solutions to increase equity in dementia care in the UK

**DOI:** 10.3389/fdgth.2024.1490156

**Published:** 2024-12-19

**Authors:** Beth Wolff, Simon Nielsen, Achilles Kiwanuka

**Affiliations:** ^1^ London School of Hygiene and Tropical Medicine, University of London, London, United Kingdom; ^2^ Brain+, Copenhagen, Denmark; ^3^ London School of Hygiene and Tropical Medicine Uganda Research Unit, Medical Research Council (Uganda), Entebbe, Uganda

**Keywords:** digital health solutions, practical guidelines, equity, dementia, UK

## Abstract

**Background:**

Digital Healthcare Solutions (DHS) are transforming healthcare by improving patients' experiences, safety and quality of care. However, despite all the proposed and observed advantages of DHS, a growing body of research suggests that these DHS are not equally accessible to all. This research aimed to assess whether equity frameworks for digital health solutions can be used to guide the development of digital health solutions to increase access to care for dementia patients in the UK and, thereafter, develop practical guidelines to guide the design of equitable DHS products to address this growing issue.

**Methods:**

A scoping review across four databases and grey literature was done to identify equity frameworks and design principles for DHS. The equity frameworks and design principles were analyzed to make recommendations on increasing equity in the product.

**Results:**

34 publications and reports met the inclusion criteria. Four equity frameworks were analyzed and one was selected. Equitable product development guidelines were created based on patient-centered design principles.

**Conclusion:**

Although DHS can increase inequity in healthcare, concrete methods and practical guidelines can minimize this if DHS developers design for maximum equity and closely collaborate with healthcare providers and end-users in product development. Future research could use these guidelines to test usability for developers and investigate other equitable approaches like institutional barriers to adoption.

## 1 Introduction

The provision of health care in the 21st century is challenged by increasingly constrained resources and evolving population demographics. To mitigate these challenges, public and private healthcare providers are digitalizing the delivery and monitoring of their care to increase access and ease the care costs.

In 2020, the World Health Organization (WHO) presented its Global Strategy on Digital Health 2020–2025 and described digitization of healthcare as “ … the field of knowledge and practice associated with the development and use of digital technologies to improve health” (1). The term “digital technologies” encompasses a broad range of care options and tools, including Digital Health Solutions (DHS). To summarize the US Food & Drug Administration's (FDA) definition, DHS are a broad category of products or tools delivered by mobile devices such as smartphone applications, telecommunication tools, or digitalized versions of previously analog tools (2). These tools offer patients, caregivers, and practitioners the ability to receive, support and provide treatment through means not dependent on physical presence or interaction (2).

DHS are transforming healthcare by improving patients' experiences, safety and quality of care (3). However, a growing body of research suggests that these DHS solutions create inequities in access and usage for providers and users (4). As equity of care is an essential metric within public health, it is critical to understand the evidence for inequity in DHS development and implementation and its contributing factors (5, 6).

The WHO defines health equity as “ … the absence of unfair, avoidable or remediable differences among groups of people, whether those groups are defined socially, economically, demographically, or geographically or by other dimensions of inequality (e.g., sex, gender, ethnicity, disability, or sexual orientation). Health equity is achieved when everyone can attain their full potential for health and well-being” (7). Health inequities are partially attributed to the Social Determinants of Health (SDoH), the conditions in which people are born, grow, live, work, play and age (8). To understand the inequities within digital health, researchers and policymakers use the SDoH to create a subset of determinants of health, called Digital Determinants of Health (DDoH), to describe the unique factors that lead to inequity in digital healthcare (9–11). These DDoH reflect a growing digital divide that separates those who have access to digital health and those who do not, prompting researchers and policymakers to urge the inclusion of equity principles in the design and implementation of DHS before inequities become embedded in digital health (6, 7, 11, 12).

Much as research suggests that these DHS solutions create inequities, whether DHS create or reduce social inequities in health is context-dependent on factors like social-economic status (13). Other approaches that have been used to reduce health inequity are: using implementation science in projects (14), drafting policies that reduce social disadvantage (15) improving health literacy of citizens (16), having structural interventions that reduce access to healthcare services (17).

Dementia is a disease area increasingly targeted by DHS developers due to the rise in the burden and inadequate resources of care in many countries. In the UK alone, the number of people living with dementia is projected to increase by 80% over the next 20 years, from 900,000 in 2020 to almost 1.6 million in 2040; while the cost of care for dementia is projected to increase from £37 million in 2020 to £94 million over the same period (18). Currently, the cost of dementia care is disproportionately borne by family members and social caregivers, with 40% of care provided unpaid by families and loved ones, 45% provided by social care [defined as care provided by private caregivers or community programs and not the National Health Service (NHS)] and 14% provided by the healthcare system in 2019 (18).

This paper will focus on a dementia treatment developed in the UK called Cognitive Stimulation Therapy (CST). CST is traditionally delivered through 14 or more in-person group sessions by trained therapists to a mixed group of 5–7 Persons with Dementia (PwD) (19, 20). Clinical evidence for the effectiveness of the therapy in increasing cognitive function and quality of life shows it to be more cost-effective than other standards of care when considering cognitive and quality of life measures. Thus, the National Institute for Health and Care Excellence (NICE) recommended CST for treating mild to moderate dementia (21). However, the in-person delivery requirement limits CST care offerings due to the dependency on the limited number of trained therapists and available spaces in a given geographical area.

Multiple digital solutions are being developed for CST to increase the availability of the therapy. This paper will assess a product developed by a digital health provider, Brain+, that digitalizes the delivery of CST to ensure high-quality delivery of CST and ease the workload required in each session by the trained therapist, which later will also be available for home use by caregivers and PwD (22). The company plans to generate evidence on cognitive improvements for PwD, validate the clinical outcomes, certify according to the medical software regulations, and apply for reimbursement from the NHS and other providers. The NHS is yet to formalize a DHS-specific reimbursement pathway. The National Institute for Health and Care Excellence (NICE) published a breakthrough approval in May 2022 of a DHS for the first-line treatment of insomnia, thereby also acknowledging the benefits and validity of the treatments (23). Since then, several DHS have seen reimbursement through value-based procurement models through the NHS.

DHS are expected to improve healthcare services provision by increasing access and lowering the costs of treatments and care (24). This paper argues they must be developed intentionally to minimize barriers to adoption and usage. To this end, this research explored the existing literature to assess whether equity frameworks for digital health solutions can be used to guide the development of digital health solutions to increase access to care for dementia patients in the UK. The researchers identified frameworks and design principles developed to reduce digital inequity. These principles were then consolidated into practical product development guidelines to guide the design of DHS, using the Brain+ CST Assistant app as a use case, to create the most equitable solution possible.

## 2 Methods

A scoping review approach was undertaken to answer the research question. Our protocol was developed using the Population, Intervention, Comparison and Outcome (PICO) model and published in PROSPERO with the number CRD42024411139 (25). The population was DHS developers in dementia, the intervention was equitable DHS design principles and equity frameworks, the comparison was current DHS development principles, and the outcome was a method for equitable development.

### 2.1 Information sources and search strategy

This study utilized two search strategies: one for the identification of equity frameworks for DHS development and the second for design principles for dementia DHS in the UK.

#### 2.1.1 Search strategy 1: equity frameworks for DHS development

Systematic reviews, research articles and grey literature from non-government organizations and other policy institutions were considered relevant for the search. The authors searched PubMed, Directory of Open Access Journals and Wiley Online Library as the main databases. The internet was also searched to capture grey literature from sources like the WHO that have developed equity frameworks.

The key concepts used in the search were “Equity” and “Digital health”. Alternative search terms used were “Equity framework*”, “Equity guideline*”, “Equity model*”, “Digital health solution*”, “Digital health tool*”, and “e-health*”.

#### 2.1.2 Search strategy 2: design principles for dementia DHS in the UK

The same databases were used with slightly more emphasis on grey literature searches as the design principles were more likely to be found on organizational websites. The key concepts used in the search were “Guideline*”, “Development”, “Digital health solution*”, “Dementia”, and “UK”. Alternative search terms were “Requirement*”, “Standard*”, “Design”, “Creation”, “Digital health”, “Digital health tool*”, “e-health solution*”, “Alzheimer's disease” and “Great Britain”.

### 2.2 Inclusion and exclusion criteria

For search strategy 1, only articles published after 2010 were included because of the relatively new nature of digital health technologies and the newly emerging awareness of inequities in the field. The post-COVID-19 pandemic time was especially relevant due to the significant increase in the level of digital care during the pandemic.

For similar reasons, search strategy 2 also included articles published after 2010 because of the relatively new nature of digital health technologies and the newly emerging approach of person-centered care for Dementia and Alzheimer's disease in the UK.

The authors excluded non-English, non-German or non-Danish papers because the authors could not translate the other languages.

### 2.3 Study selection process

The studies and frameworks identified through the search process were assessed for their relevance and fit for purpose using predetermined selection criteria, the date of publication of the article or framework and its relevance to the quickly evolving developments in care and technology, the presentation of an actual framework and not only a discussion about elements in a framework, the inclusion of practical implementation and usage evidence vs. general theoretical discussion, data results used for the article or framework based on real-life assessments or evaluations vs. theoretical assessments of literature or theory, articles or frameworks that focus on technology developed directly for patient use, articles or frameworks that focused on dementia or elderly care, and frameworks that included the SDoH and DDoH.

### 2.4 Data abstraction

The articles selected from the literature search were categorized into relevant topics in an MS Excel file for easy reference and final selection in the project. The identified equity frameworks were analyzed to assess if any one of the frameworks was comprehensive enough to cover all elements or if it was necessary to combine multiple frameworks into one comprehensive framework. The frameworks were assessed based on the feasibility of usage in the target population, in this case, DHS developers. A comprehensive analysis of co-design principles and dementia care requirements was completed. The results were used to identify the appropriate design method for the population and recommendations for how to access the population for design input.

Following the analysis, the equity framework findings were merged with findings from the co-design principles into a comprehensive Equity Mapping Table. This was done in a table to allow for easy mapping and visualization of the equitable development parameters.

A full description of the main features of the product related to the technology requirements, the accessibility by users, the usability requirements, and the description of different modules and user interfaces were provided by Brain+ in an Excel file for analysis and comparison. The data were made available in a password-protected file that was stored on the authors' computers and destroyed afterward to comply with confidentiality requests from the company. The Equity Mapping Table parameters, together with the features of the Brain+ app, were then used to design disease-agnostic practical guidelines for DHS developers to improve equity in DHS in their product.

### 2.5 Ethical considerations

A waiver for ethical approval to conduct the study was obtained from the London School of Hygiene and Tropical Medicine Research Ethics Committee after assessing whether there was any risk of conducting the research. Administrative approval was also obtained from Brain+ to conduct the study.

## 3 Results

### 3.1 Literature search

**Figure 1:**
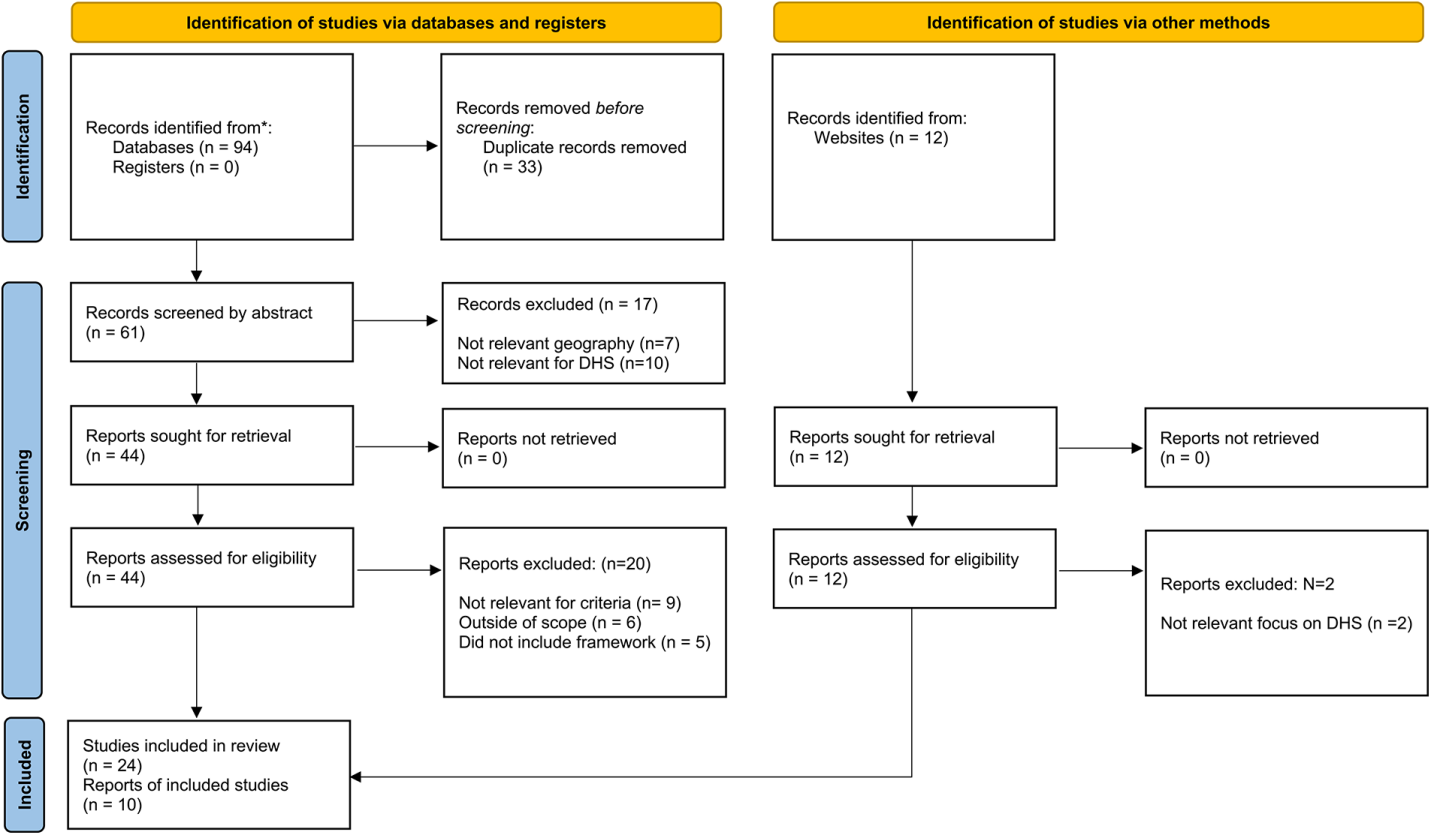
PRISMA flowchart outlining the data collection process.

The literature search resulted in 34 articles, websites and publications that were relevant for inclusion after completion of the selection process. An overview of the full selection process is depicted in Figure 1.

### 3.2 Summary of All included publications

The 34 publications selected for inclusion covered the main themes relevant to the research question which were: Digital Health, Equity and Equity Frameworks; and Guidelines for the development of DHS for dementia in the UK.

#### 3.2.1 Equity in health care, the SDoH and DDoH

Two publications (6%) defined equity in health in practical and societal terms. This was relevant to the research question as it provided real-life contexts directly affected by the digitalization of healthcare. 6 publications (18%) clearly defined the SDoH and DDoH, their relevance for healthcare and healthcare provision, and how they relate to access and effectiveness of a healthcare intervention. The definitions were relevant to the research question by providing a foundation for issues of equity in healthcare, explanations of the impact of digital tools on health, and how digital will relate to other societal elements affecting healthcare.

Fourteen publications (41%) defined the elements and criteria of digital health and what equity means within digital health. Additionally, these publications discussed the emergence of digital health as a new service and tool within general healthcare provision, the implications of digital on access to care, documented issues of digital inequity, and the growing “digital divide” within society. These articles were relevant to the research question as they provided concrete definitions of digital health and digital health solutions, provided evidence for inequity within digital health, and discussed the importance and impact of inequity in digital health on general healthcare services.

#### 3.2.2 Design principles and methods used to design digital products specifically for dementia

Seven publications (21%) discussed design principles for software and other consumer products, presented products designed specifically for PwD and their caregivers, and identified challenges in developing products for people with dementia. These publications provided detailed descriptions of design principles, including the benefits and disadvantages of each principle and focused on principles specifically for patients. The publications specifically related to designing products for dementia described real-life use cases and applications of products and analyzed the challenges and benefits for the DHS developers.

Six publications (18%) discussed co-design and innovation centers for dementia care in the UK, commonly called Living Labs, which allow DHS developers to interact with PwD and their caregivers. Additionally, the publications described the interaction process, and goals of Living Labs, and discussed the benefits and challenges for successful DHS design cases. These publications provided real-life examples of co-design practices and described how developers can interact with users in realistic settings. Finally, the publications provided practical guidance for developers on integrating the learnings into agile and iterative design processes, thereby successfully combining common practices from healthcare services and digital product design.

#### 3.2.3 Key results for the study

Out of the 34 included records, only 13 articles were highly relevant to address the research question from the two search strategies. A paper was deemed relevant if it presented a framework, a real-life assessment, provided practical and not theoretical guidance and included either the SDoH or DDoH. The relevant articles for search strategy 1 and search strategy 2 are presented in Tables 1,2 respectively.

**Table 1:** Key results for search strategy 1—equity frameworks for DHS development.

Study Details	Presentation of a framework	Real-life assessment	Practical guidance, not only theory	Include SDoH/DDoH
(3)	Yes, the author mirrored the hierarchy of SDoH elements with comparable DDoH elements	No, various examples were given of where the framework could be used, but no live testing	Yes, practical guidance provided from implementation science, business development, human-centered-design and agile methodologies	Yes
(5)	Yes, the authors integrated the existing health equity framework with new digital equity parameters	Yes, illustrated a case study of a patient during COVID-19 adopting new digital tools for disease management	No, the article focused on the importance of a framework, described an example of inequity in digital health and concluded that equity and digital health always must be linked	Yes
(10)	Yes, authors expanded an existing health disparities framework with digital equity relevant factors	Partially, a case study for remote patient monitoring was used as a model but not a real use case	Yes, developed a model illustrating how to apply the framework to a case study. Framework gave practical guidance on areas to consider for equity	Yes
(12)	Yes, authors integrated a sociological model for health equity to different levels of policy affected by DDoH	Yes, applied the model to a German COVID-19 warning app and assessed effectiveness using model parameters	Partially, discussed the practical possibilities of the model but did not offer concrete guidance to health providers or DHS developers	Yes

**Table 2:** Key results for search strategy 2—design principles for dementia DHS in the UK.

Study Details	Practical guidance, not only theory	Real-life assessment	Developed for patients	Dementia Focus
(26)	Yes, a literature review of 20 articles assessing the effectiveness of co-design through interviews, surveys, focus groups and other data-gathering means	Yes, a literature review that included studies and real-life interventions	Yes, all studies were focused on digital healthcare solutions using tech developed for patients and caregivers	No
(27)	Partially, a meta-analysis of results from 28 RCTs studying the impact of digital health solutions on depression in caregivers of people with dementia. The RCTs studied effects but did not give practical guidelines	Yes, all RCTs included interventions with caregivers in daily life situations	No, analyzed RCTs for caregivers and not for patients, but included the perspective of patients on how they might be affected by caregiver mental health and their ability to give care	Yes
(28)	Partially, a review of case studies, observational studies, reviews and commentaries on key design concepts and methods	Partially, case studies and observational studies included real-life usage	Yes, included an assessment of patient-led design case studies	No
(29)	Yes, a review of 12 studies including patients and caregivers focused on testing practical usage; and guiding on how to use a Lab	Yes, the 12 studies included patients, caregivers in real-life settings and trialing of new solutions	Yes, all studies included patients and/or their caregivers	Yes
(30)	No, a description of Living Labs, why they are created, and the differences in their approaches	Partially, descriptions of real-life Living Labs	No, referral to technology	No
(31)	No, a review and links to Living Labs throughout Europe	Yes, an overview of actual existing labs	Yes	No
(32)	Yes, provides feedback on how to use a Lab from an actual Living Lab in the UK	Yes, describes the tech used and process used to test	Yes, described how Lab engages patients and caregivers and pairs them with tech developers/solutions	Yes
(33)	Yes, provides recommendations based on a case study of a real-life Living Lab and the interventions tested there	Yes, study based on actual testing at a Living Lab	Yes, reviewed tech developed at the Lab with patients	Yes
(34)	Yes, describes an actual process and methodology used to design and test an app	Yes, uses an actual build of an app as a case study	Yes, included patients and caregivers in the design process	Yes

## 4 Discussion

### 4.1 Systemic inequity in healthcare

Sociology and other disciplines studying societal inequity have revealed an inherent inequity existing in healthcare systems like that found in other societal systems (11). Equity in healthcare differs by country, with more centralized healthcare systems in strong social welfare countries, such as Australia, Canada, and the western European countries, usually ranked more equitable than the decentralized and smaller welfare state countries like the United States (35–37). In 2021, the UK ranked 4th in two of the studies, achieving a rather high level of health equity compared to the EU and global rankings (36). The data show that regardless of its social welfare system, a level of inequity exists in every country that cannot be overlooked when assessing services offered within the system. As digital is an integrated part of every system, the same factors leading to inequity in any social structure will also apply to digital and that will affect access and a person's relationship to DHS. There are two key sets of factors that provide a basis for structural analysis of digital inequity, the Social Determinants of Health (SDoH) and the Digital Determinants of Health (DDoH).

### 4.2 The social determinants of health

Common amongst health equity discussions in the literature was a reference to the SDoH. SDoH were first published in 1999 and they have expanded as new understanding of social influences on health has emerged (38). Examples of SDoH are the environment in which people are born and raised, where they live, their education, work environment, access to healthcare, level of income and social status, and the conditions in which they age. The premise of the SDoH is that a person's health is significantly impacted by their environment and social situation and that people of different social statuses will often experience different health outcomes solely due to the difference in their social status. Social determinants are potentially more important than genetics and biology in influencing health and could account for 30%–55% of health outcomes (8).

### 4.3 The digital determinants of health

As the use of digital tools expands within healthcare services, similar levels of disparities are arising, resulting in what is often referred to as *the digital divide*. The digital divide refers to the “gaps between individuals, communities, or larger populations of people that do or do not have access to critical technologies, including health technology” (3). Digital divides exist amongst different geographies, races, genders and are caused by the affordability of technologies and services, and digital literacy, smart-phone use, internet use patterns, amongst others (3).

The significant impact created by the digital divide has prompted researchers and policymakers to identify DDoH that can be linked to SDoH (9, 11). DDoH factors lead to inequity resulting from the digital divide and impact health outcomes on various levels. Examples of DDoH are an individual's level of digital literacy (defined as how familiar they are with technology and how able they are to use digital tools), their access to technology, access to the internet, access to healthcare providers using tech, and policy makers' willingness to invest in digital infrastructure (11).

To better model the relationship between the DDoH and SDoH, the factors related to digital equity can be categorized into *individual* (unique to an individual's situation), *societal* (impacted by the society where an individual lives), and *structural* (macro level) factors.

### 4.4 Equity frameworks for digital health

#### 4.4.1 Framework type 1: positive correlation between the DDoH and SDoH

**Figure 2:**
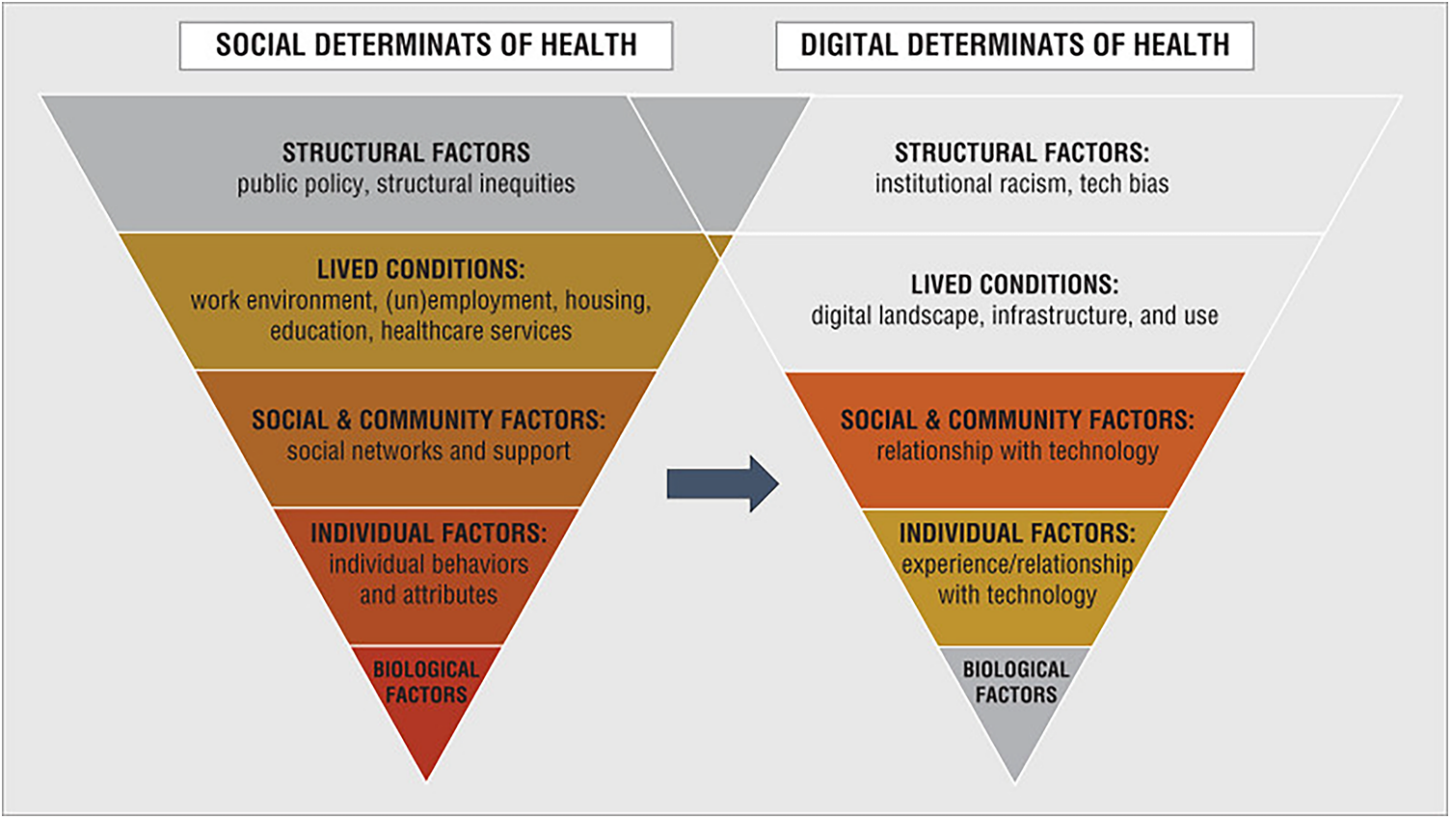
Social and digital determinants of health (3).

**Figure 3:**
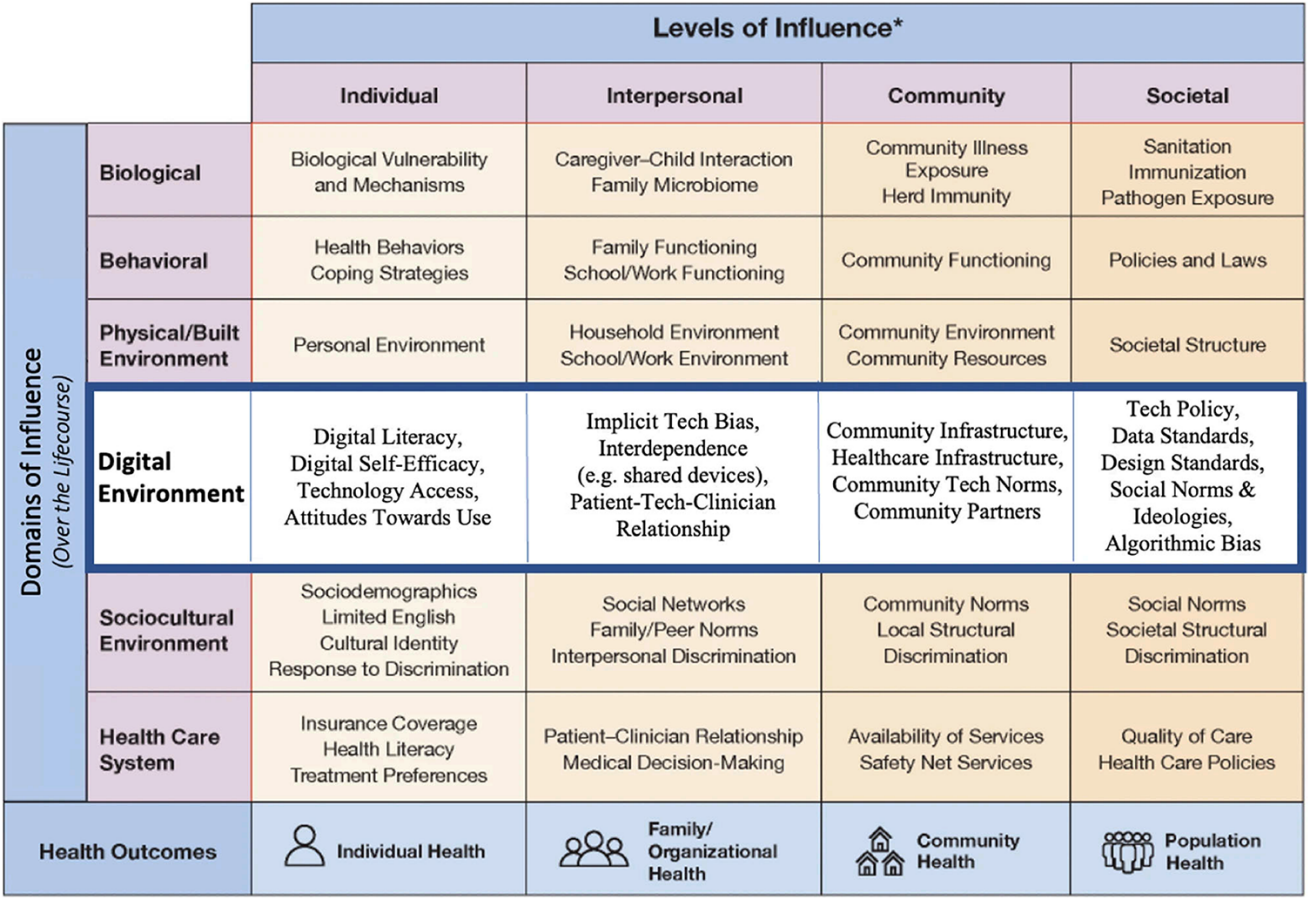
National institute on minority health and health disparities research framework expanded for digital health equity (10).

Two identified frameworks described a positive correlational relationship between the SDoH and the DDoH and linked the individual, societal, and structural DDoH factors unique to digital health solutions to the same factors unique to the SDoH (3, 10). These frameworks are the *Social and Digital Determinants of Health* framework, as depicted in Figure 2 (3) and the *National Institute on Minority Health and Health Disparities Research Framework Expanded for Digital Health Equity* as shown in Figure 3 (10). For analysis purposes, these will be labeled as Framework Type 1.

In these frameworks, the unique DDoH factors were listed either in parallel to the SDoH or integrated into an SDoH framework to create a comprehensive mapping of the different digital factors and how they relate to the overall social factors. Neither publication directly quantified the relationship between the SDoH and the DDoH; however, they provided evidence that SDoH and DDoH are subject to similar external impacts and that a greater level of general health inequity could be correlated to a greater level of digital health inequity. This correlation between the two areas of healthcare equity is useful when assessing which levels or areas of health to prioritize if attempting to lower overall inequity and avoid replicating current health inequities in new digital health offerings.

#### 4.4.2 Framework type 2: hierarchical and dependent relationship

**Figure 4:**
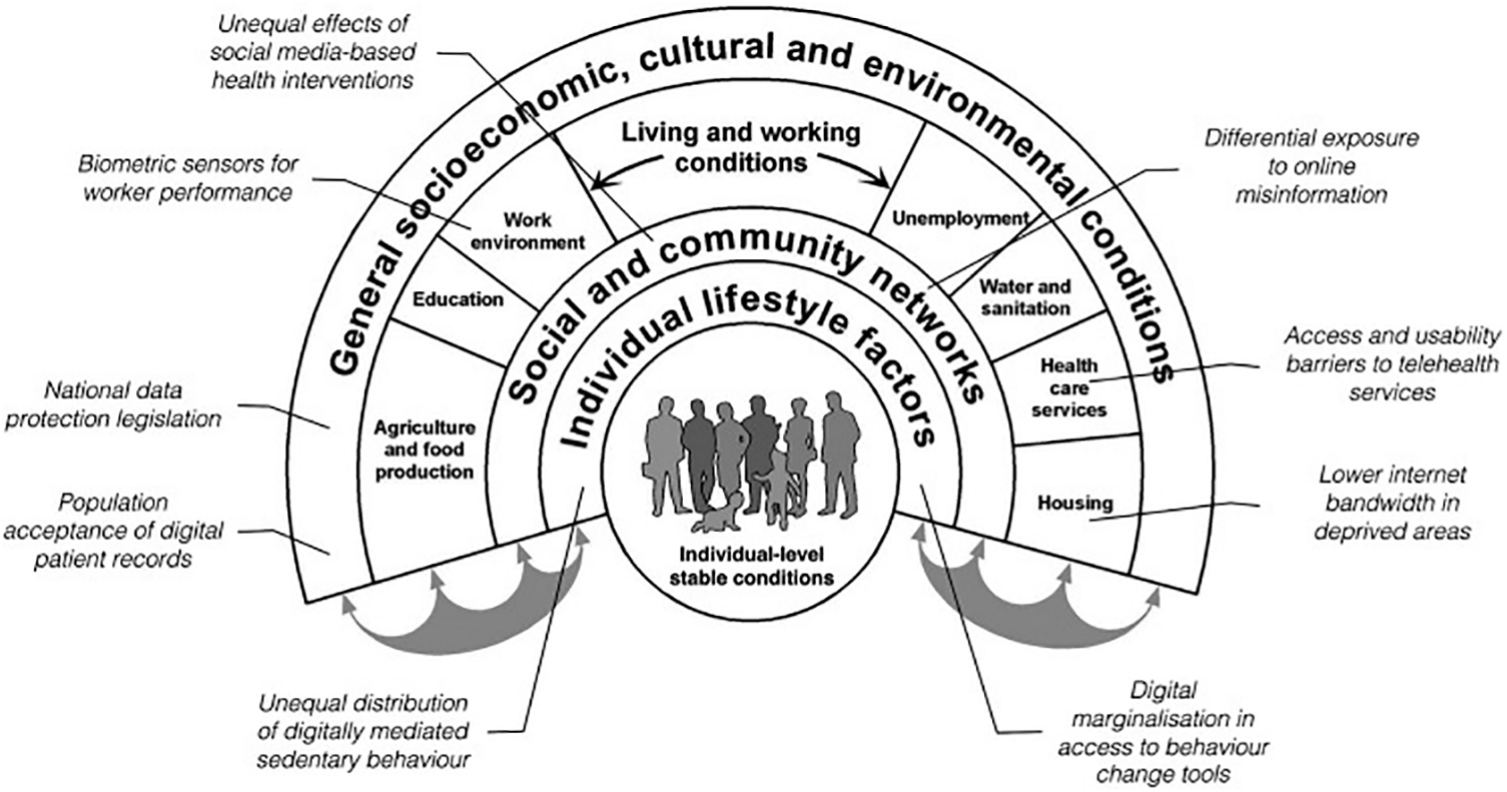
The rainbow model with examples of digital entry points for health inequality (12).

In a different approach, Jahnel et al., adapted the Dahlgren and Whitehead *“Rainbow” model* of health inequities to show the hierarchical relationship between different levels of society and how those are impacted by digital technology, see Figure 4 (12). This will be referred to as Framework Type 2. Published in 1991, Dahlgren and Whitehead created the Rainbow model as part of a WHO Europe initiative to identify strategies to reduce health inequities in the region (39). They suggested four policy levels: national/international, work and living conditions, community, and individual, where different initiatives could be aimed to positively impact health equity.

Jahnel et al., added digital as an additional factor that permeates every level to illustrate that, “ … From today's perspective, health inequities increasingly depend on digital determinants” (12). This inclusion in policy levels elevates digital from being just one element in a larger system of inequities that shift over time to being a key influencer on inequity and a target in itself to reduce inequity. It also suggests that people, healthcare systems, and policymakers cannot choose to opt out of the digital world without risking more inequity for those under their responsibility of care. This is especially relevant for factors like an individual's level of digital literacy, the community's willingness to enable the adoption of digital tools, and the establishment of adequate internet and data privacy infrastructure to ensure access and safety for all citizens.

#### 4.4.3 Framework type 3: causal relationship

**Figure 5:**
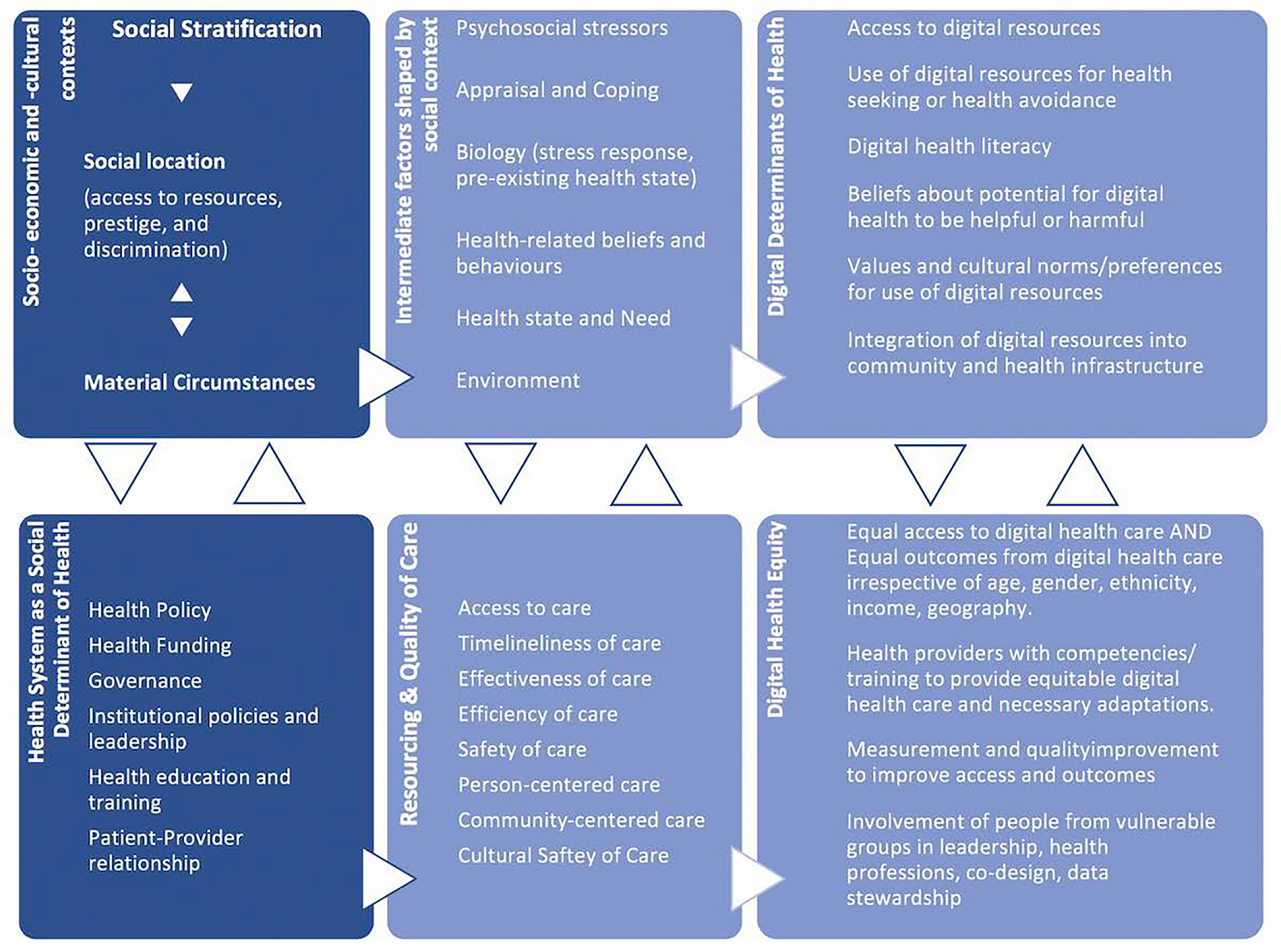
Digital health equity framework (5).

The third type of framework, Framework Type 3, as shown in Figure 5, is the *Digital Health Equity Framework* developed by Crawford and Serhal which attempts to illustrate the complexities that result in digital health inequities (5). Developed as one of the first frameworks for digital health equity, it is based on an earlier model outlining the process of allocation of a person to a social location, based on unequal distribution of power and resources. This social location then dictates, amongst other things, a person's level of access and attitude towards care, their psychological stressors, how they cope with health, and their current health needs. The authors predict that these factors will adversely affect a person's attitude towards DHS and ultimately their ability to access and use digital health solutions.

Combined, these factors impact the level of access to DHS, the quality of care, and the representative level of end-user involvement in the design of digital healthcare in different social areas. As these two paradigms do not exist independently of one another, rather they interact causally, then the factors relevant to DDoH impact digital health equity and how the system manages digital health equity becomes a DDoH. To apply the framework to DHS means that a person's social status and how the healthcare system treats them creates an emotional and physical environment that affects how they view and manage the requirements of digital tools, which then ultimately impacts their level of digital health equity.

### 4.5 Framework most relevant to equitable DHS development

The selection of the framework most relevant for DHS developers was based on two criteria: the content of the framework and the anticipated ease of adoption by solutions developers who potentially are not familiar with public health principles but rather have backgrounds in business, software development, or science. Based on these criteria, *Framework 3* offered a comprehensive set of factors to measure the impact of one factor on another, however, it is potentially too complex a framework for DHS manufacturers as it requires knowledge of the interlinking dynamics of healthcare and society to accurately understand and assess the impacts and use in the design of a relevant product. *Framework 2* showed the dependent factors related to digital equity and illustrated the wider health context for developers to consider, however, it was potentially also too complex for developers who might lack full oversight of the health policy level or the ability to fully assess the impact of information about a person's work or living conditions.

Based on the criteria, one of the types in *Framework 1*, the *National Institute on Minority Health and Health Disparities Research Framework Expanded for Digital Health Equity* was the most relevant to guide equitable digital health development. The framework lists the different elements of the digital environment that affect the usage of DHS and further separates the elements based on digital domains and levels of influence so a developer can easily identify where their product could face resistance in access and which domains of influence are available when developing for different populations. In addition, the framework can be used “as is” and does not require more complex calculations or measurements of impact by one domain or another.

### 4.6 Gathering and understanding user needs

Integrating the equity framework into a design process will require a deep understanding of the end-user's needs and their social/economic/health/digital affinity circumstances. Due to the multi-dimensional nature of digital, DHS development will bring together separate industries: technology, software, and healthcare, which often do not share common competencies and processes on how to understand the needs of the end-users (26). Development and design principles for software and technology often follow methods like agile and sprints, which focus on fast cycles, quick learning, and testing concepts in rapid repetition. Healthcare-driven principles often include surveys, observational studies, and longer and more complex intervention assessments. These differences in methods risk a misalignment in timing, purpose, and means of insights gathering, as observed in a case study of a digital application to be used for dementia (34). In this case, the design team was not able to include end-users in the needed number or frequency of meetings due to the vulnerable nature of the PwD and caregiver populations. Gaps in common terminology and “language” between the developers and healthcare professionals were also found, which led to confused communication and the incorporation of incorrect feedback. The challenges of digital health usage amongst vulnerable populations were also observed by researchers in Finland studying the adoption of digital health during the COVID-19 pandemic (40). They found that cognitive decline and inadequate guidance or support about the product could prevent older adults from engaging with the technology.

As a result, healthcare services providers are adopting the requirements of technology solutions as part of their implementation procedures for DHS and are expecting DHS developers to engage in traditional healthcare practices to understand patient needs in their design processes (27). There are various design approaches that developers can follow to understand the complexities of the disease and patient populations they are developing for: User-Centered Design, Person Based Design, Human Centered Design, Patient-Centered Design and Patient Led Design (28). The different approaches have unique benefits and challenges depending on the use case of the DHS (28). DHS developers should evaluate the most relevant design approach based on the objectives above and select the most fitting method.

The following Table 3 is a summary of a review by Duffy et al. (28), that outlines the different design approaches available to developers.

**Table 3:** A comparison of the advantages and challenges of 5 key design approaches.

Design Approach	Advantages	Challenges
UCD (User Centered Design)	Relevant for all categories of DHS, engages a large research community in a user-validated process allowing for direct input	Difficult to define the end-user and to align differing user preferences. Mostly based on qualitative feedback with small sample sizes that limit the rigor of research.
PBD (Person Based Design)	Empathically guided, psychoanalytical approach to assess improvement in well-being, well suited to change behavior. Also includes passive users not just active end-users	Specific metrics on behavioral change may not transfer to other DHS types. Methods based on a specialist psychoanalytic approach could exclude other partners in the process not trained in the method
HCD (Human Centered Design)	Adopted by prominent health care providers, recognized/standardized internationally. Combines anthropology and sociology design under a “social innovation” approach appealing to a diverse audience	A combination of many approaches from different fields of research potentially creates too much complexity for DHS developers. The need to gather input from a wide range of users and partners could impede agile working methods and timelines
PCD (Patient-Centered Design)	Focused on “patient” instead of “user” to create closer alignment with health care needs. Aims to empower patients to take control of their health and care, thereby increasing relevance and adherence to DHS	The danger of oversimplifying the complexity of health safety and clinical practices by placing patients as experts. Risks placing priority on “pop” culture attitudes and trends, not aligned with healthcare guidelines, could lead to misdiagnosis or incorrect treatment
PLD (Patient Led Design)	Based on machine learning provision of large data sets for better analysis and triangulation of preferences. Allows patients to lead the design aligned with personalized and accessible healthcare	As with PCD, the approach also limits input from a broader healthcare environment and could favor patient preferences over established healthcare methods and outcomes standards.

Using the Brain+ CST product for dementia as a use-case, the following analysis was conducted using the criteria in Table 4.

**Table 4:** Criteria for selecting the appropriate design approach.

Design Descriptor	Product Requirement	Relevant Design Approach
Who is the target user profile?	Therapists using CST to treat PwD	User-Centered Design + Patient-Centered Design
How will the product be used?	In group sessions with 4–8 PwD	User-Centered Design + Patient-Centered Design
Should the product be usable for other disease areas?	No, the product is based on a therapy only relevant to dementia	User-Centered Design + Patient-Centered Design
Is the product used to treat or manage the disease?	The product will deliver CST, an evidence-based treatment for dementia	Patient-Centered or Person Based Design
Is the product adaptable to the user?	Yes, the product can be customized by the therapist as part of treatment planning	User-Centered Design + Patient-Centered Design
Must the product adhere to standardized design principles?	Yes, it will be approved under Medical Device Software (UK MDR) regulations with standardized design and usability processes	Any design approach able to be adapted to medical device regulations

Based on this analysis, the preferred design approach for the Brain+ product was a combination of User-Based-Design and Patient-Centered-Design, with the opportunity to use Patient-Led-Design for customization features and Person-Based Design for features aimed at changing behaviors as part of the treatment. This approach allows both the therapists and PwD to be heard and give their input and would be relevant and desirable for any disease area when developing a PDT as the product is highly specific for that patient population.

This analysis is supported by a literature review on design principles for People-Centered Care Digital Solutions (26). People-centered care is increasingly accepted as the standard for dementia as it focuses care on equity principles such as respect for the individual, understanding of the individual and humanity, and customization of care to individual needs. Sanz et al., found that co-design principles such as Patient-Centered- and Patient Led Design, in which the patients and/or caregivers are the focus and can participate in the design, lead to the greatest success in product acceptance and usage (26). These findings are also supported by research done by Lyles et al., on multilevel determinants of digital health equity that shows co-design methods are best at integrating user needs into the products as they treat the patients as the experts in their needs instead of relying on the DHS developers to be the experts (9). Both studies argued that equity in DHS development is best achieved through close interaction and involvement with the patients.

### 4.7 The use of living labs to facilitate the co-design processes

A scoping review by Verloo et al., on how to best create co-design environments for DHS development in dementia identified “*living labs*” as a way to bring developers and patients/caregivers together in a mutually beneficial cooperative and creative setting (29). Living labs are “user-centric innovation environments built on everyday practice and research, with an approach that facilitates user influence in open and distributed innovation processes engaging all relevant partners in real-life contexts, aiming to create sustainable values” (30).

Ethics constraints and costly market research processes can be a barrier for DHS developers and living labs offer a more cost-effective interaction with end-users. The European Union has established a European Network of Living Labs, of which the UK is a member (31). The objective is to encourage and connect living labs to enable co-design with end users in multiple industries. In the UK, the Centre for Collaborative Innovation in Dementia is part of the European Union network and an example of a living lab creating equitable solutions for PwD (32). The center works together with the local mental health NHS Trust and uses methods such as the dementia-specific quality of life assessment (DEMQOL) to measure the effectiveness of the intervention on people with dementia (33). The UK DRI (Dementia Research Institute) Care Research & Technology Centre is another example of a living lab that brings together clinicians, scientists, engineers, designers and people affected by dementia to test technology for smart homes of the future that would allow dementia patients to remain in their homes and receive technology-assisted care (41). Living labs should be considered by DHS developers as a viable way to combine their design methods with those of the healthcare environment to avoid misunderstandings, misalignment, and incorrect product design input.

## 5 Practical guidelines for developing equitable DHS

**Figure 6:**
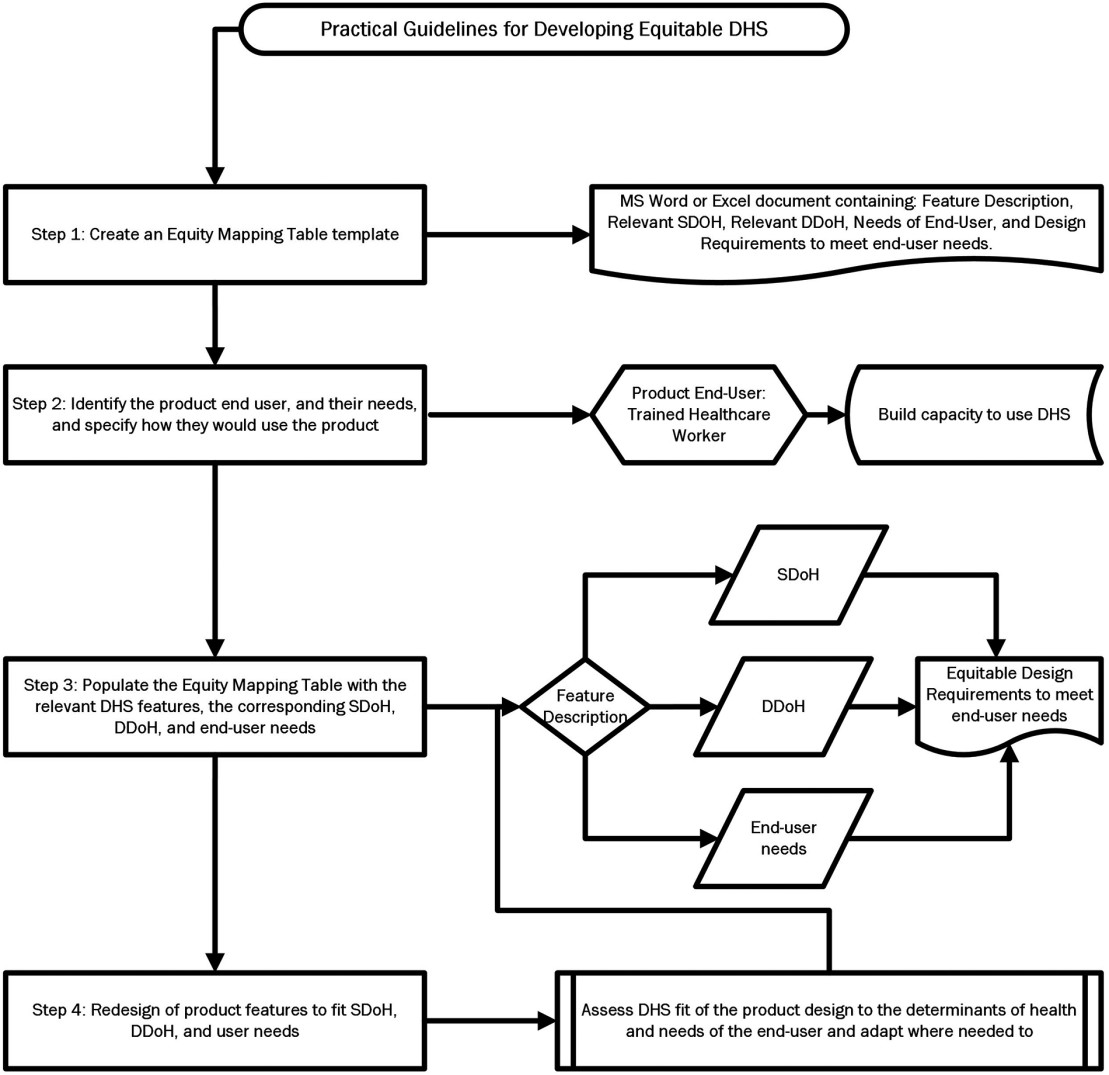
Practical guidelines for developing equitable DHS.

Using the Brain+ CST app as a use case, this paper suggests the following steps to utilize the selected *Framework 1* equity framework and the discussed relevant design principles to guide a process for equitable product development as depicted in Figure 6.

*Step 1: Create an Equity Mapping Table template*

This table can be made in a simple Excel file or Word document and should contain the following categories: Feature Description, Relevant SDOH, Relevant DDoH, Needs of End-User, and Design Requirements to meet end-user needs.

*Step 2: Identify the product end user, and their needs, and specify how they would use the product*

The end user would be trained CST therapists of various educational backgrounds such as occupational therapists, dementia care therapists, and psychologists. Input gathered from a co-design process run by the company revealed that the therapists are not always highly experienced technology users based on their background and current job requirements, that the individual or center providing the tablet or computer to run the app might not have the financial means to purchase the latest and newest devices, and the end user might not have the familiarity or trust in digital technology to support a therapy that is highly personalized and based on close human interaction.

The therapists would use the product during weekly in-person 2 h sessions, either 2 or 3 times a week, when the therapy would be administered to a group of 4–8 PwD, most likely in a care home or community center. The digitalized version of the therapy would be available to the therapist on a tablet or online on a computer. The therapist would select different topics for the session and would use the device to guide the group through the exercises, which consist of pre-programmed questions using appropriate visual and auditory stimuli.

*Step 3: Populate the Equity Mapping Table with the relevant DHS features, the corresponding SDoH, DDoH, and end-user needs*

Use the framework to determine the relevant SDoH and DDoH for the selected DHS features, the needs of end-users related to the selected Feature requirement which were gathered in a co-design process should be added to the appropriate column in the Table.

*Step 4: Redesign of product features to fit SDoH, DDoH, and user needs*

In this final step, the developer should assess the fit of the product design to the determinants of health and needs of the end-user and adapt where needed to resolve gaps in design that would have excluded populations from use or prevented intended outcomes of the solution.

An example of equity mapping is presented in Table 5. The intended outcomes of the equity mapping process are to alert the DHS developers to the social and digital barriers to their product's access, as well as to provide a structured process to resolve inequitable feature design with equity requirements and recommendations for design change.

**Table 5:** Equity mapping table for brain+ App.

Feature Description	Relevant SDoH	Relevant DDoH	Needs of end-user	Design requirements to meet end-user needs
The product can be easily operated by the intended user on a tablet and computer	Individual	Digital Literacy, Technology Access, Attitudes Towards Use	Low level of tech skills required to use the product, product compatible with older versions of tablets or computers, comprehensive tutorials within the product to guide users without extra support	Currently, the product is available on iOS 11 and Android 4.4 or newer versions. The product is available in the Chrome, Firefox, and Edge browsers. Consider developing for older iOS and Android versions. Consider adding Safari to browser options to allow for broader access on iOS versions.
The patient is presented with content that is culturally adapted to their language, age, cognitive ability, nationality	Interpersonal	Interdependence, Patient-Tech-Clinician Relationship	Product is immediately customizable by a therapist within a personal session with PwD, content is usable as-is with minimal intervention from the therapist to meet a wide range of PwD backgrounds, and highly relevant content builds trust and collaboration between PwD and therapist to maximize treatment outcomes	The product currently allows non-developers to update and curate content; the product allows users to customize their settings and experiences. Recommend including a co-design process to maximize relevance and relatability to PwD and their lives. Potentially include insights from co-design in-app features to increase therapist understanding of patient group
The patient is presented with content that is culturally adapted to their language, age, cognitive ability, nationality	Community	Community Infrastructure, Healthcare Infrastructure, Community Tech Norms	The product reflects an understanding of local cultural norms, the healthcare practices and interactions of a given country or area, the relationship between CST therapists and PwD/caregivers, the level of digitalization within community care centers	Product design must include a comprehensive “localization” process, including end-user participation, to approve the language and cultural references, ensure relevance for local care practices, confirm the usability of the tech provided, and support appropriate care and therapy objectives
The device complies with all applicable requirements and guidance	Societal	Tech Policy, Data Standards, Design Standards, Social Norms & Ideologies	The product can be run on existing devices approved by healthcare centers or individuals without the need to buy new devices, the product complies with all GDPR and local UK data privacy standards, the product can be used with or without live internet access	The product will be approved as a medical device software (UK MDR) that requires compliance with data privacy and security standards. Recommend gathering understanding on willingness to share data and personal information from PwD to lower the risk of alienating patients or caregivers.

## 6 Conclusion

The literature reveals consensus amongst policy leaders, healthcare providers, and academics that inequity exists in digital health and must be addressed if healthcare systems continue to digitalize. DHS developers will play a critical role in shaping the equity of healthcare in the future and should share responsibility for creating equity in return for public funding and resources. Guidelines and frameworks can focus attention on areas of inequity and can be applied in internal and external design processes to increase patient and provider influence on product development.

DHS can be developed for many diseases and thus the same design principles apply to DHS developers of other diseases. Similarly, the same principles can be applied beyond the UK to DHS developers in other parts of the world since the SDoH and DDoH are globally applicable. To develop equitable solutions, DHS developers should: understand and acknowledge the systemic and inherent inequity in the healthcare system, adopt the mindset that their digital health solutions will be subject to the same factors of social inequity that impact other health provisions, assume that the users of their products live with fundamental barriers to digital adoption based on their social situation, integrate co-design procedures as a prerequisite to their tech design, and put the users' needs and abilities above the possibilities of the tech to ensure greatest adoption and usability.

New technology must be developed and implemented with equity in focus and with an active attempt to decrease and resolve the current barriers to care. As DHS are often developed by those outside the healthcare system, they might not be aware of the systemic inequity and how it impacts the usage of digital health solutions. Therefore, developers must be cognizant of a potential correlation, dependency or causality between digital health solutions and health inequity.

Equity frameworks and design principles can help DHS developers identify individual, institutional, societal, and community-level SDoH and DDoH factors needed to understand the interdependencies between the access and usage of tech and the factors influencing overall health status and equity. Selecting the appropriate design principles and utilizing offerings such as living labs can provide the needed input for DHS developers who do not have easy access to patient populations. Finally, using *The Equity Mapping Table* as a guide can enable methodical and transparent equitable product development.

For the healthcare industry, equity requirements and frameworks should be integrated into Medical Device Regulations and other tech for healthcare regulations. Regulators and/or user communities could also co-create equity certifications for DHS developers. International policy organizations such as the World Health Organization are already discussing the need for these types of certifications and potentially will be recommending them in future policy papers and initiatives to minimize inequity in the digitalization of healthcare.

Although DHS can increase inequity in healthcare, concrete methods and practical guidelines can minimize this if DHS developers design for maximum equity and closely collaborate with healthcare providers and end-users in product development. Future research could use these guidelines to test usability for developers and investigate other equitable approaches like institutional barriers to adoption.
